# The diagnostic utility of D-dimer and other clinical variables in pregnant and post-partum patients with suspected acute pulmonary embolism

**DOI:** 10.1186/s12245-018-0169-8

**Published:** 2018-03-05

**Authors:** Hyun Choi, Dinesh Krishnamoorthy

**Affiliations:** 1 Emergency Department, University Hospital Lewisham, Lewisham High Street, London, UK; 2 St George’s School of Medicine, University of London, Cranmer Terrace, London, SW17 0RE UK

**Keywords:** Pulmonary embolism, Pregnancy, Post-partum, D-dimer

## Abstract

**Background:**

Pulmonary embolism (PE) during pregnancy remains one of the leading causes of maternal morbidity and mortality in the developed world. However, there is a paucity of high-quality evidence resulting in a lack of consensus in managing this group of patients. The aims of the study were to address the diagnostic utility of D-dimer for suspected PE in pregnant and post-partum patients and to identify any clinical presentation variables that are predictors of PE in this group of patients.

**Methods:**

A retrospective case note review of 152 pregnant and post-partum patients who underwent diagnostic imaging (ventilation/perfusion (V/Q) or computed tomographic pulmonary angiography (CTPA)) for suspected PE at a tertiary teaching hospital from 2007 to 2011 was conducted. The reference range for D-dimer was less than 0.5 mg/L as being normal. The following variables were also assessed in terms of their predictive capability for PE diagnosis in pregnancy: heart rate (HR), mean arterial pressure (MAP), shock index (SI) and A-a gradient.

**Results:**

The application of D-dimer testing for suspected PE in this study population had a sensitivity of 100% (95% CI, 73–100%), specificity of 42% (95% CI, 31–53%) and a likelihood negative ratio of 0. None of the clinical variables were significant predictors of PE according to regression analyses.

**Conclusions:**

There is supportive evidence that a negative D-dimer result is useful as a means of ruling out PE in pregnant and post-partum patients. However, we need a larger prospective observational study to collaborate the findings.

## Background

Pregnancy-related pulmonary embolism (PE) remains one of the leading causes of maternal morbidity and mortality in the developed world [1]. The risk of antenatal venous thromboembolism (VTE) is four- to fivefold higher in pregnant women than non-pregnant women of the same age [2]. Venous thromboembolism can occur at any stage of pregnancy, but the puerperium remains the time of highest risk with an estimated 20-fold increase in relative risk [3]. This is because each component of Virchow’s triad (venous stasis, hypercoagulable state and tissue trauma) is present at some stage during pregnancy [4].

The Confidential Enquiry into Maternal and Child Health (2004) in the UK identified areas of substandard care in two thirds of the cases, including failures in obtaining objective diagnoses and acting promptly in providing necessary treatment in suspected cases of VTE [1].

PE amongst pregnant and post-partum patients remains arguably one of the most difficult diagnostic challenges for clinicians. Many of the signs and symptoms of PE are similar to the manifestation of physiological changes that occur during the course of pregnancy. Radiation exposure is also a particular concern with consequent implications of carcinogenesis and teratogenesis and interruptions of breastfeeding for post-partum lactating women. Historically, the diagnosis of PE has relied on validated diagnostic algorithms, such as the Wells rule [5], for constructing the pre-test probability of the disease. The Wells rule [5] includes the clinicians’ subjective judgement of whether PE is more likely than an alternative diagnosis. New rules, such as the Geneva [6], Pisa [7], Charlotte [8] and pulmonary embolism rule-out criteria (PERC) [9] rules, contain objective items only. However, none of these diagnostic tools have been validated on pregnant patients.

Our knowledge of the risk assessment of PE is largely derived from two large prospective multi-centre trials, PIOPED I and PIOPED II, conducted in the last 20 years [10, 11]. The most widely used clinical model for PE, Wells rule [5], was extracted from relevant clinical presentation characteristics detected in PIOPED trials. However, pregnant and post-partum patients were excluded from these trials. Hence, the same clinical prediction rules do not apply to this group of patients.

The diagnostic utility of D-dimer for PE is well established in non-pregnant, low-risk patients [5]. However, its role in pregnancy is uncertain because of the substantial increase of D-dimer throughout gestational age [12, 13]. This increase is thought to be related to the rising levels of circulating fibrinogen in pregnancy, rather than due to an increase in levels of fibrin degradation products [12].

A simple management strategy is urgently required for pregnant and post-partum patients with suspected PE in order to provide a rapid and reliable diagnosis while avoiding unnecessary anticoagulation treatment and radiation exposure.

The objectives of our study were largely twofold. First of all, we attempted to address the diagnostic utility of D-dimer for PE in pregnant patients and those within 6 weeks post-partum. Secondly, we attempted to identify any clinical presentation variables that were predictors of PE during pregnancy and post-partum periods. In particular, we looked at the association between the diagnosis of PE and the median heart rate (HR), mean arterial pressure (MAP), shock index (SI) and A-a gradient.

## Methods

Consecutive pregnant and post-partum patients presenting to the emergency department (ED) or antenatal clinics with clinically suspected acute PE between January 2007 to January 2011 at a tertiary teaching hospital in London, who underwent V/Q scans, CTPAs or pulmonary angiography were retrospectively selected for data review. The post-partum period lasted for 6 weeks after the delivery of the foetus or termination of the pregnancy. Patients were identified electronically from all V/Q scans, CTPA and pulmonary angiography previously performed for suspected PE during the study period. The selected patients’ case records were then examined for the recording of their clinical presentation, D-dimer results and outcomes.

A radiological diagnosis of PE was defined by the PIOPED criteria [10, 11]—a high-probability ventilation/perfusion (V/Q) scan with no previous history of PE, i.e. ≥ 2 segmental perfusion defects (V/Q mismatch), a positive CTPA scan or pulmonary angiography.

D-dimer was measured by MDA Auto-Dimer, an immunoturbidimetric assay that utilises antibody coated latex particles. The reference range for D-dimer was less than 0.5 mg/L as being normal.

A-a gradient (kPa) was calculated from the following equation: [(Fi_O2_) × (atmospheric pressure − H_2_O pressure) − (Pa_CO2_/0.8)] − Pa_O2_ [14].

Shock index was calculated as HR/systolic BP.

The sensitivity and specificity with 95% confidence intervals (95% CI) were calculated to assess the diagnostic performance of D-dimer for PE in pregnant and post-partum patients. Positive and negative predictive values (PPVs, NPVs) were also reported with 95% CIs.

The D-dimer levels and gestation of the pregnancy at presentation were noted and compared between the two groups (non-PE and PE) using the Mann-Whitney *U* test. The clinical presentation parameters, namely heart rate (HR), mean arterial pressure (MAP), shock index (SI) and A-a gradient, were also compared between non-PE and PE groups, using the two-tailed *t* test or the Mann-Whitney *U* test (on condition of non-normal distributions).

To assess the predictive capability of the aforementioned clinical parameters for PE diagnosis in pregnancy, the univariate regression analyses were performed using Student’s *t* test. Significance was fixed at *p* value < 0.05.

All statistical analyses were performed using spreadsheet-based statistical software (StatsDirect, release 2.3.8; CamCode, Herts, England).

The study was discussed with the local research and development department who advised that formal ethics committee approval was not required for this project due to its observational nature. All data were anonymised.

## Results

Overall, 152 pregnant and post-partum patients within 6 weeks of delivery or termination of pregnancy underwent V/Q scans or CTPAs as part of their assessment for suspected PE over a 4-year period between 2007 and 2011. No pulmonary angiogram was performed on any of these patients.

Patient demographics for the study population are given in Table 1.

**Table 1:** Characteristics of the study population

Total number of women, *n*	152
Weeks of gestation at presentation, *n* (%)	
< 12	23 (15.1)
12–28	42 (27.6)
> 28	59 (38.8)
Post-partum	28 (18.4)
Age, mean (SD), years	31.4 (5.1)
Nulliparous, *n* (%)	35 (23.0)
Gravity, mean (SD)	2.9 (2.1)
Twin pregnancy, *n* (%)	3 (2.0)
Termination of pregnancy, *n* (%)	1 (0.7)
Outcomes	
In-hospital mortality, *n* (%)	0
28-day mortality, *n* (%)	0
ICU/HDU/CCU admissions, *n* (%)	8 (5.3)
Reasons for ICU/HDU/CCU admission	
Intra-abdominal bleeding following anticoagulation	1
Asthma exacerbation	1
H1N1	1
Other URTI/LRTI	2
Thrombolysis for saddle pulmonary embolus	1
Eclampsia	1
PCI following MI (CCU)	1

Data are presented as number (percentage) of women, unless otherwise indicated

*CCU* coronary care unit, *ICU* intensive care unit, *MI* myocardial infarction, *URTI* upper respiratory tract infection, *LRTI* lower respiratory tract infection, *PCI* percutaneous coronary intervention

Of 152 patients, 93 had the D-dimer assay performed as part of their assessment for PE. One hundred thirty had the HR and MAP documented in their case notes, and 110 patients had the blood gas measurements documented.

Table 2 shows a 2 × 2 table for the binary variable ‘PE diagnosis’ and the dichotomised variable ‘D-dimer test’ using a cut-off point of 0.5 mg/L that distinguished between a positive (> 0.5 mg/L) and negative (< 0.5 mg/L) test result.

- Sensitivity = 100% (95% CI, 73–100%)
- Specificity = 42% (95% CI, 31–53%)
- Positive predictive value (PPV) = 23% (95% CI, 14–36%)
- Negative predictive value (NPV) = 100% (95% CI, 87–100%)
- Likelihood ratio positive (LR+) = sensitivity/(1 − specificity) = 1/(1–0.42) = 1.72 (95% CI, 1.42–2.07)
- Likelihood ratio negative (LR−) = (1 − sensitivity)/specificity) = 0/0.42 = 0

**Table 2:** Performance of D-dimer for detecting PE in pregnant and post-partum patients

		PE diagnosis		
		Negative (0)	Positive (1)	Total
D-dimer test	Negative (0)	33	0	33
	Positive (1)	46	14	60
	Total	79	14	93

In both groups, the spread of D-dimers was not normally distributed for either set of patients (non-PE and PE) across all stages of gestation.

**Fig. 1:**
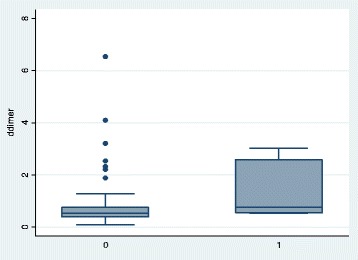
Box plot of D-dimer for patients without (0) and with PE (1)

A box plot of D-dimer for the two groups is shown in Fig. 1, and the D-dimers were compared using the non-parametric Mann-Whitney *U* test. There was a significant difference in the median values between the two groups (*p* = 0.0061).

The null hypothesis that the D-dimer variable is the same between non-PE and PE pregnant and post-partum patients was rejected.

**Fig. 2:**
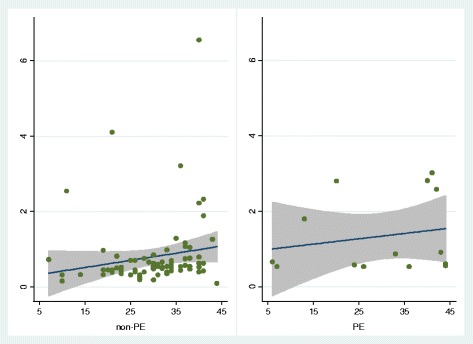
D-dimer against number of weeks of gestation and post-partum

Figure 2 illustrates D-dimer levels against week gestation for non-PE and PE groups respectively.

Despite the fact that Fig. 2 appears to show increasing linear trends throughout gestation for both non-PE and PE groups, these trends are not significant in a regression analysis.

**Table:** 

Regression results from non-PE group (79 patients):				
D-dimer	Coefficient	*p* value	95% CI	
Constant	0.236455	0.534	− 0.51644	0.989347
Weeks	0.018921	0.124	− 0.00529	0.043131
Regression results for PE group (14 patients):				
D-dimer	Coefficient	*p* value	95% CI	
Constant	0.920815	0.2	− 0.55866	2.400287
Weeks	0.014102	0.509	− 0.03101	0.059215

Table 3 illustrates the median values for non-PE and PE patients and the *p* values obtained when comparing the clinical presentation variables for the two groups. None of the presentation variables were significant at the 95% level. Only the A-a gradient was close to being significant for these data.

**Table 3:** Comparing clinical presentation variables in PE and non-PE groups

Characteristic	Median [IQR]		*p* value	Significant?
	Non-PE	PE		
Heart rate (HR)	97 [82, 110]	95 [80, 119]	0.9121	No
Mean arterial pressure (MAP)	85 [77.67, 92.67]	92 [79.33, 98.33]	0.2975	No
Shock index (SI)	0.81 [0.70, 0.95]	0.84 [0.54, 0.89]	0.6195	No
A-a gradient	1.3 [0.5, 3.1]	3.3 [2.4, 4.2]	0.0739	No

Table 4 summarises the results of the clinical presentation characteristics as predictors of PE after univariate regression analyses. None of the variables were significant predictors of PE diagnosis. However, the A-a gradient was borderline significant.

**Table 4:** Patient presentation characteristics and their association with PE diagnosis in pregnancy and the puerperium (univariate analysis)

Presentation characteristics	Odds ratio	95% CI	*p* value
D-dimer	1.52	[0.95, 2.42]	0.14
Heart rate (HR)	1.02	[0.56, 1.85]	0.65
Mean arterial pressure (MAP)	1.54	[0.89, 2.66]	0.15
Shock index (SI)	0.70	[0.33, 1.51]	0.47
A-a gradient	2.08	[0.93, 4.62]	0.081

## Discussion

Currently, there is a paucity of data that addresses the diagnostic utility of D-dimer in pregnant and post-partum populations with suspected PE. Not surprisingly, there is no clear guidance on the management of this specific cohort of patients due to the lack of evidence available.

Before our study, the only available data that directly addressed the above clinical dilemma was by Damodaram et al. [15], a retrospective case study comprising 37 women with suspected PE that underwent ventilation/perfusion diagnostic scanning. There is also a case report based on a single pregnant patient with PE [16]. Hence, our retrospective study comprising 93 pregnant and post-partum patients with recorded D-dimer levels represents the largest study sample to date.

Our study data showed that the sensitivity and specificity of D-dimer as a screening test for suspected PE in pregnant and post-partum patients undergoing emergency diagnostic imaging was 100 and 42%, respectively, while the negative likelihood ratio was 0 (Table 2). These results appear to be principally dictated by the lack of a single positive PE diagnosis when the D-dimer measure was below the 0.5 mg/L cut-off.

By contrast, Damodaram et al. [15] showed the sensitivity of 73% and specificity of 15%, respectively, while the negative likelihood ratio was 1.8. Their D-dimer cut-off point was also 0.5 mg/L. However, a moderate probability V/Q was also considered as a positive diagnostic test for PE. This is against the current guidelines [10, 11, 17]. A single case report by To et al. [16] illustrated a case of a pregnant patient with objectively diagnosed PE, but with a negative D-dimer.

Clearly, we need a larger prospective study to determine with confidence if a negative D-dimer result can safely rule out the diagnosis of PE in pregnancy as our study findings contradict those of the existing smaller retrospective study [15] and of the case report [16].

In the non-pregnant woman, a negative MDA latex agglutination D-dimer test helped to rule out VTE (both DVT and PE) in those with both intermediate and low clinical probability with the pooled sensitivity 97%, specificity 46%, +LR 1.8 and −LR 0.07 [18]. It is likely that the same D-dimer assay, as utilised in our study, could be potentially useful as a means of ruling out PE in pregnant and post-partum patients.

Our D-dimer data appeared to show increasing linear trends throughout gestation for both non-PE and PE groups (Fig. 2), but these trends were not significant in a regression analysis. This lack of significance could be due to the impact of outlying values and a lack of power attributable to the small sample size. Unfortunately, we had insufficient data to apply the results to patients in the individual stages of gestation (trimesters and post-partum).

Our data did not show any significant differences in median HR, MAP, SI and A-a gradient amongst pregnant and post-partum patients between non-PE and PE groups (Table 3). Only the A-a gradient was close to being statistically significant. None of these clinical presentation variables were significant predictors of PE during pregnancy and post-partum periods.

There is no published data that addresses the relationship between the A-a gradient and PE in pregnant and post-partum populations.

Stein et al. examined the data derived from PIOPED I for any potential diagnostic utility of an A-a gradient for PE in non-pregnant patients [19]. They found that normal values of the A-a gradient did not exclude the diagnosis of acute PE.

One needs to recognise a fundamental physiological difference in the population characteristics between those in PIOPED I and II trials [10, 11]—generally older patients with variable baseline cardio-pulmonary function vs. pregnant and post-partum patients and younger patients with healthy baseline cardio-pulmonary function. Hence, an abnormal A-a gradient could prove to be a more significant predictor of PE amongst our study cohort, compared to that of the PIOPED cohorts. Further investigation and more data are needed to assess this relationship further.

### Limitations

The study relied on the retrospective data collection with subsequent absence of some data. It is also a single-centred study with a relatively small number of participants. Nonetheless, it is still the biggest study to address the utility of D-dimer in the diagnosis of pregnancy-associated PE.

The study identified only those patients with suspected PE who underwent emergency diagnostic imaging. It is possible that some patients were discharged home from the emergency department or antenatal clinics with a normal D-dimer and no imaging but had subsequently developed PE. Such patients would not have been captured in our data if they had sought health care services elsewhere for treatment.

Finally, it is possible that CTPA or V/Q imaging were considered unnecessary in some patients but still treated for presumed PE, i.e. positive bilateral leg DVT scans or echocardiography findings suggestive of PE. The study focused on CTPAs and V/Q scans as the diagnostic imaging of choice, as this is the accepted practice in the authors’ hospital. However, it is likely that there are other accepted modes of investigation across different healthcare settings. This lack of agreement is due to the absence of consensus in the national or international guidelines.

## Conclusions

None of the patients with PE had a D-dimer below the 0.5 mg/L cut-off level, making it a potentially useful screening tool for ruling out PE with a negative D-dimer.

An A-a gradient performed better than D-dimer in predicting PE in our study cohort. However, neither of them were significant predictors of PE diagnosis in pregnancy. We need a larger prospective observational study to collaborate these findings amongst pregnant and post-partum patients.
